# Individualized margins for prostate patients using a wireless localization and tracking system

**DOI:** 10.1120/jacmp.v12i3.3516

**Published:** 2011-05-22

**Authors:** Prema Rassiah‐Szegedi, Brian Wang, Martin Szegedi, Jonathan Tward, Hui Zhao, Y. Jessica Huang, Vikren Sarkar, Dennis Shrieve, Bill Salter

**Affiliations:** ^1^ Department of Radiation Oncology University of Utah Salt Lake City UT USA

**Keywords:** margins, prostate cancer, wireless localization, Calypso

## Abstract

This study investigates the dosimetric benefits of designing patient‐specific margins for prostate cancer patients based on 4D localization and tracking. Ten prostate patients, each implanted with three radiofrequency transponders, were localized and tracked for 40 fractions. “Conventional margin” (CM) planning target volumes (PTV) and PTVs resulting from uniform margins of 5 mm (5M) and 7 mm (7M) were explored. Through retrospective review of each patient's tracking data, an individualized margin (IM) design for each patient was determined. IMRT treatment plans with identical constraints were generated for all four margin strategies and compared. The IM plans generally created the smallest PTV volumes. For similar PTV coverage, the IM plans had a lower mean bladder (rectal) dose by an average of 3.9% (2.5%), 8.5% (5.7%) and 16.2 % (9.8%) compared to 5M, 7M and CM plans, respectively. The IM plan had the lowest gEUD value of 23.8 Gy for bladder, compared to 35.1, 28.4 and 25.7, for CM, 7M and 5M, respectively. Likewise, the IM plan had the lowest NTCP value for rectum of 0.04, compared to 0.07, 0.06 and 0.05 for CM, 7M and 5M, respectively. Individualized margins can lead to significantly reduced PTV volumes and critical structure doses, while still ensuring a minimum delivered CTV dose equal to 95% of the prescribed dose.

PACS numbers: 87.53.Kn, 87.55.D

## I. INTRODUCTION

The planning target volume (PTV) as defined by ICRU 62^(1)^ is designed to account for the net effect of all possible geometrical variations and inaccuracies in order to ensure that the prescribed dose is actually deposited in the clinical target volume (CTV). In other words, the PTV includes compensation for the effects of organ and patient movement, and inaccuracies in beam and patient setup. Conventionally, both interfraction and intrafraction motion are accounted for by the PTV.

For prostate cancer, previous studies have shown that patient setup based on skin and bony landmarks may result in inadequate target coverage if insufficient margin is used^(^ ^2^ ^,^ ^3^ ^)^ since the prostate moves independently of the surrounding bones of the pelvis. In an effort to reduce the CTV‐to‐PTV margin size and yet maintain target coverage, many radiation therapy centers are employing image‐guided radiation therapy (IGRT) techniques such as ultrasound,^(^ ^4^ ^,^ ^5^ ^)^ kV,^(^ ^6^ ^,^ ^7^ ^)^ or MV^(^ ^8^ ^,^ ^9^ ^)^ imaging with or without implanted fiducials. These techniques are capable of correcting daily interfractional variations in target positioning.

These daily localizing techniques remove the geometrical uncertainties in prostate positioning associated with patient setup and interfraction movement due to such occurrences as bladder/rectal filling. The uncertainty is removed to the degree of accuracy of the system that localizes the prostate. Hence, margins only need to account for intrafraction motion, such as motion due to breathing or bowel gas movement, which is smaller than margins that account for both intrafraction and interfraction movement. In order to account for intrafraction motion, a system which tracks the prostate motion during treatment is needed. The advent of the 4D electromagnetic system (Calypso Med.Tech. Inc., Seattle, WA) for prostate positioning and tracking^(^ ^10^ ^–^ ^12^ ^)^ has made it possible to track the daily intrafractional motion of the prostate during radiation treatment without the ill effects of extra‐radiation. Therefore, intrafraction motion can be measured for a large population of patients which may, in turn, enable the study and design of margin strategies based on population or individuals. In this retrospective study, we investigated the feasibility of individualizing margins based on intrafraction motion as reported by the 4D tracking system.

In order to use individualized margins, an individual patient's motion must be studied, and a margin strategy must then be designed for each patient — an action requiring extra resources and time. The strategy of individualizing patient margin will only be worth the effort if it results in clinically significant normal structure dose reduction along with increased or equivalent target coverage.

The purpose of this work is to investigate the potential dosimetric benefits of designing patient‐specific margins for prostate cancer patients based on observation of prostate motion during the initial few treatments using a 4D localizing and tracking system for a typical fractionation regime of 1.8–2 Gy in 35–42 fractions.

## II. MATERIALS AND METHODS

### A. Calypso system overview

The Calypso Localization System (Calypso Medical Technologies, Inc., Seattle, WA) is a real‐time monitoring system which uses an array of electromagnetic coils to generate and receive a resonant response from implanted transponders (beacons). An infrared optical tracking system tracks the position of the array relative to the machine isocenter. Hence, the array location, together with the transponder locations relative to the array, yields the position of the treatment isocenter within the prostate, with respect to the machine isocenter. The display system updates itself at a frequency of 10 Hz over a field of view of 14 cm×14 cm, and is thus capable of tracking the prostate position during treatment. Several studies have assessed the the system and found it to be within submillimeter accuracy.^(^ ^13^ ^,^ ^14^ ^)^ Accuracy of transponder localization relative to the array decreases with depth. At 27 cm depth (most challenging clinical scenario), the accuracy is ± 0.27 mm, ± 0.36 mm, and ± 0.48 mm in the lateral, longitudinal, and vertical direction, respectively.^(13)^ The ability of the system to localize transponders accurately has been shown to be unaffected by transponder motion^(15)^ within reasonable limits.

### B. Clinical process

Ten prostate patients, each implanted with three transponders, were included in this study. Transponders were implanted at least one week prior to the treatment planning CT. Transponders were inserted into the prostate under transrectal ultrasound guidance by deployment through 14 gauge needles directly into the prostate gland following a lidocaine nerve block. The transponders were implanted at the apex, right mid‐base and left mid‐base. During the planning process, the three‐dimensional coordinates (x, y and z) of the transponders, and the treatment isocenter are recorded and transferred to the Calypso workstation located at the linear accelerator (linac) treatment suite.

Patients were instructed to arrive for treatment with moderately full bladders and were set up initially using skin marks and lasers. The Calypso system was then used for localization/ alignment, based on the transponders. The system displays the deviation of the prostate treatment isocenter to the machine isocenter. Pretreatment adjustment of the couch/patient was performed, such that the deviation from machine isocenter in x (RT/LT), y (Inf/Sup), and z (Ant/ Post) direction read 0±0.5 mm for the treatment isocenter. Post‐localization, the system was used to track the position of the treatment isocenter/prostate relative to the machine isocenter until the end of treatment delivery. All 10 patients were localized and then tracked for the entire duration of treatment for all fractions, for a total of 400 fractions.

### C. Individualized margin (IM) design

For the purpose of exploring the potential benefits of a patient Individualized margin (IM) strategy, a reasonable strategy for defining a patient‐specific margin needed to be identified. Through retrospective review of the patient‐specific tracking data collected for the 10 patients studied here, an initial straightforward and reasonable strategy was to utilize three standard deviations (3σ) of the first five fractions' target variation as a predictor of the full course target deviation. We note that this strategy is conceptually similar to the ‘no action level’ protocol^(^ ^16^ ^–^ ^18^ ^)^ proposed by De Boer and Heijmen in 2001.

Since the tracking data are collected after the initial localization, the mean displacement for all patients was 0±0.5 mm. Table 1 depicts the R/L, I/S, and A/P values of (3σ) of the first five fractions target motion for each patient (column set 1), whereas two standard deviations (2σ) for the full course of 40 fractions are shown in column set 2 of Table 1. In all cases, the 3σ values for the first five fractions can be seen to be equal to or greater than the full course 2σ values, thus ensuring that at least 95% of the CTV intrafraction movement post‐localization would be covered by the margin. Since the Calypso system samples the target position at a rate of 10 times per second, a requirement that 95% of the reported target positions be within the designed margin will ensure that the minimum dose to the periphery of the CTV would be 95% of the prescribed dose.

**Table 1:** Individualized margin generated for 10 patients based on 3σ for the first 5 fractions. For patients #6 and #9, the IM is greater than 5M (highlighted in red), indicating that a 5M margin is inadequate for 95% target coverage if 5M is used for treatment.

	*3 std dev for 5 fractions*			*2 std dev for 40 fractions*			*Calculated Individulized Margin (CIM)*			*Individualized Margin (IM) Used for Plan*			
*Pat#*	*lateral/cm*	*InfSup/cm*	*AntPost/cm*	*lateral/cm*	*InfSup/cm*	*AntPost/cm*	*lateral/cm*	*AntPost/cm*	*InfSup/cm*	*AntPost/cm*	*lateral/cm*	*InfSup/cm*	*AntPost/cm*
1	1.3	3.3	3.6	1.0	2.4	3.0	2.3	4.3	4.6	4	4	**5**
2	1.0	1.4	2.8	1.0	1.8	2.4	2.0	2.4	3.8	4	4	4
3	1.4	2.3	3.1	1.2	2.2	2.2	2.4	3.3	4.1	4	4	4
4	1.2	3.1	3.4	0.8	2.0	2.6	2.0	4.1	4.4	4	4	4
5	1.3	1.7	1.7	0.8	1.0	1.4	2.3	2.7	2.7	4	4	4
6	1.2	4.5	3.5	0.6	2.0	1.6	2.2	5.5	4.5	4	**6**	**5**
7	0.5	1.3	1.2	0.7	1.0	1.2	1.5	2.3	2.2	4	4	4
8	0.9	1.5	2.1	0.6	1.2	1.4	1.9	2.5	3.1	4	4	4
9	2.4	4.0	5.3	1.6	2.6	3.6	3.4	5.0	6.3	4	**5**	**6**
10	0.9	1.4	1.4	0.6	0.9	0.9	1.9	2.4	2.4	4	4	4

The calculated individualized margins (CIM) were calculated based on three standard deviations of the tracking data from the beginning time of the 1st beam to the ending time of the last beam for all fractions. The mean value for tracking data for all fractions for all patients was less than 0.3 mm. The formula used for calculation is shown in Eq. (1) and can be seen to also include the inherent uncertainty of localization with the Calypso system.

(1) $$\text{Calculated}\;\text{Individualized}\;\text{Margin}\;(\text{CIM})=3\sigma +\sqrt{{\text{Localization}}^{2}+{\text{Optical}}^{2}}$$

where Localization = residual error resulting from localizing the transponders with the array. 0.5 mm (used for this study based on accuracy values reported by Balter et al.^(13)^), and Optical = residual error resulting from positioning the array with the optical camera (0.5 mm was used for this study).

Table 1 (column set 3 and 4) presents the individualized margins calculated and used in this study for each of the 10 patients based on the 3σ strategy described above. The delineation of the target in our study was based on RTOG 415, where prostate was equal to the gross tumor volume (GTV) as well as the CTV. Hence, we imposed a minimum patient‐specific margin limit of 4 mm for the IM, based on published pathology data reporting that a 3–5 mm margin would encompass 99% of all known tumor (extra prostatic extensions) in the 376 specimens studied.^(19)^ Therefore, the IM is actually the CIM with an imposed minimum margin of 4 mm (i.e., if a CIM is 4 mm or greater, then it is used as an IM; if the CIM is smaller than 4 mm, then 4 mm is used as IM). If the CTV includes extra prostatic extension or if the physician is comfortable, then a minimum 4 mm margin does not need to be imposed. In that instance, the CIM will be equal to the IM.

### D. Dosimetric comparison between IM, 5M, 7M, and CM plans

Having identified an effective methodology for determining a reasonable patient‐specific margin, a set of treatment plans of various margin strategies for comparison for each patient was created. A margin of 10 mm in all directions and 7 mm posteriorly from the CTV was used to create what we referred to as the “conventional margin” (CM) PTV. Seven mm uniform margin (7M) and 5 mm uniform margin (5M) plan sets were also explored, as these are margins commonly used by centers which perform some form of image‐guided treatment delivery.^(20)^ Lastly, a patient‐specific IM plan using the strategy described above was created.

Six field static gantry angles — at 40°, 100°, 150°, 210°, 260°, and 320° — intensity‐modulated radiation therapy (IMRT) treatment plans were generated for all four of the previously described margin strategies (i.e., conventional, 7 mm uniform, 5 mm uniform and patient‐specific IM), based on IMRT planning guidelines outlined in RTOG protocol 0415. Identical dose constraints were used for all plans.

To characterize the potential benefits of an IM strategy, dose volume histograms (DVHs), mean, minimum, and maximum dose to the bladder, rectum, CTV and PTV for the four margin strategies described above were compared. The dose to 2 cc (D2cc) and dose to 10 cc (D10cc) were also compared. NTCP for rectum was calculated using the Lyman Kutcher‐Burman model. The values used to calculate rectal NTCP are based on meta‐analysis of the results of four studies as given by Michalski et al.^(21)^ and is shown in Table 2. Due to the lack of consensus and lack of data for input parameters for bladder NTCP calculation in recent literature, only gEUD values are calculated for bladder.

(2) $$gEUD={\left(\frac{1}{N}\sum_{i=1}^{N}D_{i}^{a}\right)}^{1/a}$$

where *N* is the number of voxels encompassed by the contour of the anatomical structure, *a* is the normal tissue specific parameter which describes the dose volume effect, and $D^{i}$ represents the dose to ith voxel. In this study, *a* was assigned the value of 1, classifying the bladder as a parallel structure.

**Table 2:** Parameters used for NTCP calculation.

*Critical Structure*	n	${\text{TD}}_{50}$	m	*End point*
Rectum	0.09	76.9	0.13	RTOG ≥ grade 2 rectal toxicity

Abbreviations: ${\text{TD}}_{50}$ = dose (Gray) required for complication probability of 50%; m=mid‐point slope; n=volume effect exponent

## III. RESULTS

As seen from Table 1, for the 10 patients evaluated, our data suggest that even a 5 mm uniform margin may not be adequate for some patients (see patient # 6 and patient #9; values highlighted in red).

**Figure 1:**
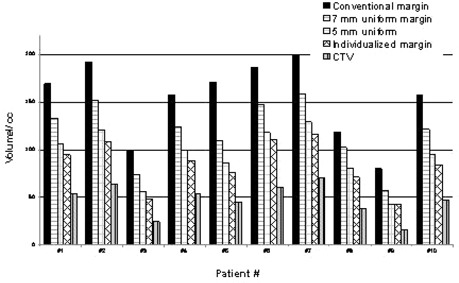
Volumes of the PTV with different margin strategies for all 10 patients and the respective CTVs (in purple). The IM provided average reductions of PTV volume of μ=80.0 cc, Range 41.1–113.9 cc; μ=33.8 cc, Range 14.4–43.7, and μ=9.3 cc, Range −0.7–12.7, relative to the conventional margin, 7 mm, and 5 mm margin, respectively.

Figure 1 summarizes the volumetric relationship of the four different margin strategies relative to the CTV. The mean absolute volume reduction in PTV with IM compared to CM, 7M and 5M is 59.6, 33.8, and 9.3 cm^3^, respectively. The IM can generally be seen to create a significantly smaller PTV volume than the CM or even 5M strategies, while still ensuring 95% isodose coverage of the target.

Table 3 presents critical structure dosimetric data for each plan by patient, and shows that the mean dose to the rectum for the IM plan is reduced by an average of 9.8%, 4.8%, and 2.5% of prescribed dose of 75.6 Gy across all patients relative to CM, 7M, and 5M plans, respectively. Similarly, Table 3 also shows that IM plan mean bladder dose is reduced by an average of 14.9%, 6.1%, and 2.6% of prescribed dose relative to the CM, 7M, and 5M plan across all patients. For the IM plans relative to the 5M plans, reductions in mean rectal and bladder doses as large as 10.2% and 6.0%, respectively, are seen for certain patients.

**Table 3:** Differences in mean doses (Gy) for rectum and bladder for all 10 patients, for the CM, 5M, and IM margin sets.

*Mean*				*Rectum*							*Bladder*			
*Dose /Gy Patient #*	*CM*	*7M*	*5M*	*IM*	*CM‐IM*	*% Difference 7M‐IM*	*5M‐IM*	*CM*	*7M*	*5M*	*IM*	*CM‐IM*	*% Difference 7M‐IM*	*5M‐IM*
1	30.9	26.7	26.8	26.3	6.1	0.6	0.6	20.0	14.5	12.9	12.2	10.3	3.0	1.0
2	39.0	38.0	35.8	34.8	5.6	4.3	1.3	39.4	33.7	30.3	28.0	15.1	7.5	3.1
3	28.5	26.6	23.7	23.0	7.3	4.7	0.9	27.9	23.3	18.9	17.9	13.3	7.2	1.3
4	33.0	30.8	28.1	26.3	8.8	5.9	2.3	35.1	30.5	26.0	23.9	14.8	8.8	2.7
5	46.8	36.0	36.3	32.6	18.8	4.5	4.9	43.0	29.6	26.2	25.5	23.1	5.4	0.9
6	40.5	33.8	30.4	30.2	13.6	4.7	0.3	55.2	43.8	38.9	38.7	21.9	6.8	0.4
7	37.9	35.2	33.3	32.7	6.8	3.3	0.8	35.2	30.5	27.2	25.1	13.4	7.1	2.8
8	42.3	39.3	37.6	29.8	16.5	12.5	10.2	44.3	39.4	37.9	34.9	12.5	6.0	4.0
9	43.5	41.0	40.0	38.9	6.2	2.8	1.5	19.8	13.7	16.5	12.0	10.3	2.2	6.0
10	38.5	35.7	33.7	32.5	8.0	4.3	1.6	31.2	25.0	22.8	20.1	14.6	6.5	3.5
				Average	9.8	4.8	2.5					14.9	6.1	2.6
				Min	5.6	0.6	0.3					10.3	2.2	0.4
				Max	18.8	12.5	10.2					23.1	8.8	6.0

The maximum dose to the rectum was reduced by an average of 5.1%, 1.8%, 0.7%, whereas the maximum dose to the bladder was reduced by an average of 6.7%, 2.5%, 0.9% relative to the CM, 7M, and 5M plan, as shown in Table 4. The critical structure maximum dose generally occurs close to the border of the PTV and the critical structure. Since dose is prescribed to the PTV and there is an overlap of PTV and critical structure for all three margin strategies, the maximum doses are similar for all three margins strategies. Hence, the average reduction in maximum dose is smaller when margins are decreased, as shown in Table 4.

**Table 4:** Differences in maximum doses (Gy) for rectum and bladder for all 10 patients for the CM, 5M, and IM margin sets.

*Maximum*				*Rectum*							*Bladder*			
*dose/ Gy Patient #*	*CM*	*7M*	*5M*	*IM*	*CM‐IM*	*% Difference 7M‐IM*	*5M‐IM*	*CM*	*7M*	*5M*	*IM*	*CM‐IM*	*% Difference 7M‐IM*	*5M‐IM*
1	94.0	83.9	86.9	83.8	13.4	0.1	4.1	96.6	86.3	87.3	85.2	15.1	1.5	2.8
2	78.3	78.2	77.0	76.9	1.8	1.7	0.1	79.5	78.4	77.6	76.7	3.7	2.2	1.1
3	77.5	76.5	76.9	76.0	1.9	0.6	1.2	77.4	76.6	76.8	75.9	2.0	0.9	1.2
4	76.4	75.7	75.1	74.7	2.2	1.3	0.6	77.3	77.2	76.6	76.4	1.2	1.1	0.3
5	78.2	78.0	76.7	76.7	2.0	1.6	0.0	78.0	77.8	76.7	76.5	1.9	1.8	0.2
6	86.0	82.2	77.5	77.0	11.9	6.9	0.7	87.1	81.8	77.7	77.2	13.1	6.1	0.7
7	80.5	77.5	76.7	76.7	5.1	1.1	0.0	85.9	79.6	78.8	78.4	10.0	1.6	0.5
8	86.4	82.6	80.1	80.1	8.3	3.3	0.0	89.3	83.9	81.8	80.3	11.9	4.7	2.0
9	79.0	78.0	77.6	78.0	1.4	0.0	0.5	80.1	78.7	77.2	77.5	3.5	1.6	−0.5
10	78.9	77.2	76.8	76.3	3.4	1.2	0.6	80.4	79.9	77.6	77.1	4.3	3.6	0.7
				Average	5.1	1.8	0.7					6.7	2.5	0.9
				Min	1.4	0.0	−0.5					1.2	0.9	−0.5
				Max	13.4	6.9	4.1					15.1	6.1	2.8

While the mean critical structure dose reductions for the IM strategy relative to the 5 mm strategy may seem somewhat modest, it is worth noting that: a) there are patient‐specific instances of much larger improvements, and b) that these critical structure dose improvements are achieved while still guaranteeing 95% coverage of the PTV, versus the 5 mm strategy (which was seen in two out of ten patients studied here to be inadequate with regard to coverage).

The D10 cc to the rectum was reduced by an average of 13.7%, 7.9%, 3.4%, whereas the D10 cc to the bladder was reduced by an average of 17.6%, 9.9%, 4.2% relative to the CM, 7M, and 5M plan, as shown in Table 5. The D2 cc to the rectum was reduced by an average of 6.8%, 3.7%, and 1.8%, whereas the D2 cc to the bladder was reduced by an average of 7.2%, 3.6%, and 2.6% relative to the CM, 7M, and 5M plans.

**Table 5:** Dose 10 cc (Gy) for rectum and bladder for all 10 patients for the CM, 5M, and IM margin sets.

*D10cc*				*Rectum*							*Bladder*			
*(Gy)/Patient #*	*CM*	*7M*	*5M*	*IM*	*CM‐IM*	*% Difference 7M‐IM*	*5M‐IM*	*CM*	*7M*	*5M*	*IM*	*CM‐IM*	*% Difference 7M‐IM*	*5M‐IM*
1	85.1	77.6	76.0	71.9	17.4	7.6	5.4	89.6	76.6	70.1	64.3	33.5	16.3	7.7
2	75.1	74.6	72.2	72.3	3.7	3.1	−0.1	76.8	76.1	73.7	73.6	4.2	3.3	0.1
3	75.1	73.1	65.5	60.1	19.8	17.1	7.1	74.4	74.4	71.2	69.4	6.6	6.6	2.4
4	72.0	72.9	70.2	68.5	4.6	5.8	2.2	73.3	68.7	62.5	58.2	19.9	13.8	5.6
5	73.4	72.3	70.9	69.6	5.1	3.7	1.8	75.0	71.7	67.1	65.4	12.7	8.4	2.2
6	80.8	73.0	67.3	65.3	20.5	10.2	2.6	85.8	79.8	73.1	71.8	18.6	10.6	1.8
7	74.5	71.2	66.5	58.6	21.0	16.6	10.4	80.6	78.0	76.5	70.4	13.5	10.1	8.1
8	67.7	63.4	59.6	57.6	13.4	7.6	2.7	83.0	72.5	64.7	63.4	26.0	12.1	1.7
9	78.1	63.6	60.1	59.1	25.1	5.9	1.2	75.1	62.2	62.3	54.7	26.9	9.9	10.0
1077.1	73.2	72.7	72.5	6.1	1.0	0.3	78.7	73.8	70.0	68.0	14.1	7.6	2.6	
			Average	13.7	7.9	3.4				Average	17.6	9.9	4.2	
			Min	3.7	1.0	−0.1				Min	4.2	3.3	0.1	
			Max	25.1	17.1	10.4				Max	33.5	16.3	10.0	

**Figure 2:**
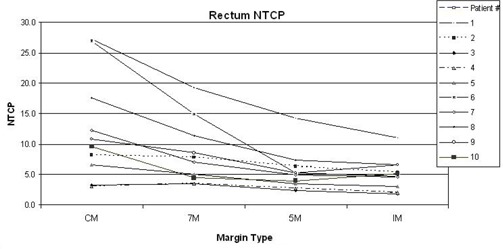
The decreasing trend in rectum NTCP is clearly seen for the 10 patients.

The trend in NTCP values for rectum is clearly seen in Fig. 2 to decrease with use of the IM strategy relative to the CM, 7M, and 5M margin strategies. The average reduction in NTCP for rectum is 10.3%, 4.6%, and 4.3% for 7M, 5M, and IM, respectively.

**Figure 3:**
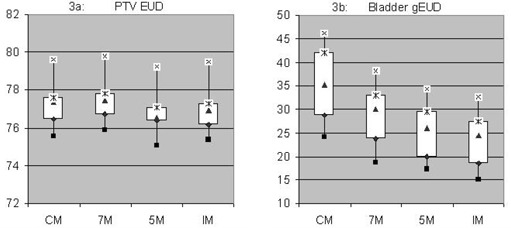
Effective uniform dose (EUD) for the PTV for all four margin strategies for all patients (3(a)) is within 2% of each other; the decreasing trend in bladder generalized effective uniform dose (gEUD) from the conventional margin (CM) to individual margin (IM) is shown in 3(b).

PTV EUDs for all 10 patients are shown in Table 6 and Fig. 3(a). There are insignificant differences in the PTV EUDs, as all plans were normalized such that the minimum dose to the PTV is 72 Gy. Bladder gEUDs are seen in Fig. 3(b) to decrease with use of the IM strategy relative to the CM, 7M, and 5M margin strategies, again indicating an advantage to using the IM strategy.

**Table 6:** The equivalent uniform dose (EUD) in Gy for the PTV.

*EUD/Gy*	*PTV*			
*Patient* *#*	*CM*	*7M*	*5M*	*IM*
1	82.5	82.5	82.0	82.5
2	77.6	77.4	76.8	76.6
3	75.1	76.0	76.5	75.7
4	76.9	77.1	74.4	75.8
5	76.2	76.6	76.0	76.0
6	77.6	7.6	77.2	76.8
7	76.3	77.9	76.4	77.3
8	79.1	79.5	79.2	79.4
9	77.4	77.7	76.6	77.1
10	77.4	76.2	76.3	77.1
AVERAGE	77.6	77.9	77.2	77.4

## IV. DISCUSSION

In this study, a 95% target coverage was chosen and hence settled with a margin based on 3σ. Based on the coverage criteria, one can choose one's own criteria or design of the margin. The CTV and GTV (prostate only) are identical, and we chose a minimum CTV to PTV margin of 4 mm. If a CTV, which includes areas at risk for microscopic disease extension, is contoured, a 4 mm margin need not be imposed and margin reduction can be more dramatic.

The results of our study imply that even a 5 mm margin may not be adequate for some patients. We note that this differs from results published in a retrospective dosimetric study by Li et al.^(20)^ where a 2 mm or greater margin was concluded to be adequate for 95% target coverage for most patients.

In that study, the dose distribution was translated, based on the motion of the prostate. Therefore, if the minimum PTV dose or prescribed isodose line (dosimetric margin) is not tightly conformed to the PTV, then the target coverage is less sensitive than predicted by the margins. Gordon and Siebers^(22)^ introduced the concept of dosimetric margin distribution (DMD), and proposed that the DMD and CTV–PTV margin be evaluated together to determine the sensitivity of a treatment plan to motion. However, current planning systems are not equipped to provide the user with DMDs and, as a result, DMDs may not be a feasible evaluation tool for routine treatment planning. Therefore, CTV–PTV margin is still the preferred and conservative approach of ensuring target coverage, though it may seem less tolerant to motion.

We believe that individualizing margin even in a clinic which uses Calypso is beneficial for several reasons. Firstly, if a clinic typically employs a large margin (i.e., 7 mm or greater), then individualizing the margin will certainly reduce the margins for most patients and, therefore, reduce toxicity. For clinics that regularly utilize smaller margins, our study shows that for certain patients, a 5 or 6 mm margin may not be adequate; hence, individualizing the margin is an alternative to gating which unduly increases treatment time. Whatever scheme (e.g., 2σ, 3σ, etc.) or criteria (e.g., 90%, 95%, 100%, etc.) coverage is used, studying the initial tracking data and creating an individualized margin is definitely beneficial, as it optimizes the margin for each individual patient.

As for clinics that have target localizing capacities but not tracking and gating tools, this study shows that a 5 mm margin may not be adequate to ensure a 95% target coverage.

The effort (if IM is implemented with our strategy) involves analyzing five days of tracking data and then rerunning the treatment plan. This extra effort is only worthwhile if the difference in the IM plan compared to the initial plan is clinically significant. If one were to assume that significant clinical benefit is achieved when NTCP values differ by 5% or greater, then our data show that clinical significance is greatest when the initial margin is large (see Fig. 2 and Table 5). Although the benefit of creating an IM generally reduces when a 5 mm margin is used for the initial plan, it may still be worth running an IM plan as some patients with 5 mm initial margins may still benefit from it, as shown for patient #3, #7 and #10. By simply observing the tracking data, one may also discover that the 5 mm margin is not adequate.

## V. CONCLUSIONS

For 10 patients and 400 treatment fractions evaluated, a strategy has been devised of defining patient individualized margins based on target motion observed during the first 5 fractions of treatment. This strategy is shown to lead to significantly reduced PTV volumes, while still ensuring a minimum delivered CTV dose of 95% of prescribed dose and equivalent EUD over the entirety of the treatment course. Furthermore, reduced bladder and rectal doses, manifested as valuable reductions in bladder and rectal NTCP, were also seen for all patients studied. We observed that a uniform margin of 5 mm may not be adequate for some patients to achieve 95% target coverage, thus highlighting the need to address planning of each patient individually.

Based on this work, the design of patient‐specific margins for prostate appears to be feasible, and dosimetrically and radiobiologically advantageous.
